# Editorial: Metastasis: From Cell Adhesion and Beyond

**DOI:** 10.3389/fonc.2019.00214

**Published:** 2019-04-02

**Authors:** Vasiliki Gkretsi, Triantafyllos Stylianopoulos

**Affiliations:** ^1^Cancer Biophysics Laboratory, Department of Mechanical and Manufacturing Engineering, University of Cyprus, Nicosia, Cyprus; ^2^Biomedical Sciences Program, Department of Life Sciences, School of Sciences, European University Cyprus, Nicosia, Cyprus

**Keywords:** metastasis, cell adhesion, lymph nodes, epithelial to mesenchymal transition, triple negative breast cancer, cluster of circulating tumor cells, solid stress, stiffness

## Metastasis

Metastasis is a complex multistep process during which cancer cells within a tumor dissociate from one another, migrate, and invade through surrounding tissues to finally enter the circulation or the lymphatic system, being thus transported to other sites of the body where they establish a new metastatic tumor. Many different approaches have been followed so far to study this process and find ways to prevent it. The collection of articles in this Frontiers Research Topic depicts exactly that.

## Cell Adhesion

Firstly, a pivotal role in the metastatic process is played by the cell-extracellular matrix (ECM) adhesion proteins as well as their interaction with actin cytoskeleton. Gkretsi and Stylianopoulos provide a concise review of the recent literature on important determinants of the cell's adhesome at cell–ECM adhesion sites that affect its invasive properties. Multiple protein-protein interactions define this adhesome linking the ECM directly or indirectly with the actin cytoskeleton (1, 2) and downstream effectors such as RhoGTPases (3) that collectively coordinate metastasis-related cellular processes. Furthermore, ECM accumulation within the tumor often leads to desmoplasia, an intense fibrotic response, causing tumor stiffening. Stiffening in turn, adds a biomechanical perspective to the whole concept of tumor growth and metastasis (4, 5). In that regard, Gkretsi and Stylianopoulos also emphasize the importance of keeping stiffness in mind when developing *in vitro* model systems.

## The Mechanical Component

Adding to the biomechanical aspect of metastasis and tumor growth, Kalli and Stylianopoulos, define the concepts of stiffness and solid stress in tumors, as so far it is not clear whether matrix stiffness and solid stress are interrelated or if they have distinct roles in tumor progression. Pointing out that increased solid stress and stiffness are two distinct biomechanical abnormalities of the tumor microenvironment, they present a review of the different effects of these two parameters on the behavior of cancer and stromal cells. They also review and compare the *in vitro* experimental approaches that have been employed so far to analyze the effect of stiffness and solid stress providing a useful guide for similar studies.

## Triple-Negative Breast Cancer Metastasis and Metastasis to the Lymph Nodes (LN)

Along the same lines, Neophytou et al. focus on one of the most desmoplastic types of cancer, breast cancer, and triple negative breast cancer (TNBC), in particular, and provide a thorough analysis of the molecular mechanism involved during epithelial-to-mesenchymal transition (EMT), as well as the genes activated in this aggressive cancer type during the different stages of metastasis (metastasis promoting genes and metastasis suppressors). Moreover, they discuss recent advances on TNBC treatment, at the preclinical level, using agents that remodel tumor microenvironment and enhance the effects of chemotherapy delivery as well as advances emerging from novel molecular targets.

As with TNBC, in many cancer types, the first sites of metastasis for the original tumor are lymph nodes (LN). In fact, LN metastasis has been associated with worse prognosis although the mechanism is still vague. Jones et al. provide herein, an overview of the seeding, growth, and dissemination of LN metastases based on recent literature. Emphasis is given on how tumor cells and their secreted molecules decrease anti-tumor immunity and promote tumor growth in the LN.

## Clusters of Circulating Tumor Cells

Tripathi et al. focus on another aggressive type of breast cancer, which is also desmoplastic, inflammatory breast cancer (IBC). In this type of cancer, metastasis occurs not only through circulating tumor cells (CTCs) but rather via the generation of CTC clusters. CTC clusters may be rare and are thought to retain some epithelial characteristics, as they do not undergo a complete EMT, but account for more than 90% of metastases. Tripathi et al. based their work on a theory suggesting that the more hierarchically organized a physical system is, the more adaptable it can become. Thus, in the research article presented in this special issue, Tripathi et al. use the cophenetic correlation coefficient (CCC) to quantify the hierarchical organization in terms of gene expression of two different gene sets. They show that indeed high CCC, of both collective dissemination-associated genes and the IBC-associated genes, is associated with higher metastatic relapse rate in breast cancer patients.

## A High-Throughput, Functional Technique for Assessing Cancer Cell Invasion Potential

Interestingly, in a more applicable point of view, the research article by Weitz et al. introduces a novel high-throughput, functional method for assessing cancer cell invasion potential. This method takes advantage of the biophysical changes occurring during metastasis that enable a cancer cell to invade the surrounding tissue. Using this technique, prostate, and bladder cancer cells are labeled with a fluorescent calcium dye and imaged during stimulation with low-intensity focused ultrasound; invasive cell lines exhibit calcium elevation which is not true for non-invasive cells (Weitz et al.). Thus, this method provides a means of assessing tumor invasion potential which could prove useful in cytology studies and ultimately improve clinical management (Weitz et al.).

## Intratumoral Immune Cytolytic Activity

Last but not least, Roufas et al. provide us with a different view of dealing with metastasis focusing on immune checkpoint blockade therapy. Contrary to the approach taken by most anti-cancer immunotherapies, immune checkpoint blockade aims at blocking immune responses by inhibiting immune suppressor molecules, thus awakening the cytotoxic T lymphocytes from dormancy and enabling them to kill the cancer cells they infiltrate (6). Here, Roufas et al. conduct a comprehensive meta-analysis to evaluate the intratumoral immune cytolytic activity (CYT) in different cancer types, as judged by the expression of toxins granzyme A (GZMA) and perforin 1, and investigate differences between primary and metastatic tumors (data obtained from The Cancer Genome Atlas and Genotype-Tissue Expression project databases). They show that the cytolytic index among other associations with tumor-infiltrated immune cells promotes evasion from immunosurveillance in certain cancers Roufas et al..

## Concluding Remarks

In this research topic, we presented a collection of articles focused on fundamental processes of cancer cell metastasis, such as cell-ECM adhesions, EMT and LN metastasis as well as on upcoming research fields including the effects of biomechanical factors, the use of analytical and statistical tools and experimental techniques to further understand and characterize the invasive and metastatic potential of tumors.

## Author Contributions

All authors listed have made a substantial, direct and intellectual contribution to the work, and approved it for publication.

### Conflict of Interest Statement

The authors declare that the research was conducted in the absence of any commercial or financial relationships that could be construed as a potential conflict of interest.
